# Social media use by health professionals: To do or not to do?

**DOI:** 10.4102/safp.v62i1.5008

**Published:** 2020-01-28

**Authors:** Lushiku Nkombua

**Affiliations:** 1 Department of Family Medicine, Faculty of Health Sciences, University of Pretoria, Pretoria, South Africa

In August 2019, the Health Professions Council of South Africa (HPCSA) released booklet 16, entitled *Ethical Guidelines on Social Media*.^1^ It is clearly stated in the booklet that healthcare professionals need to be aware that potential risks are involved in the sharing of information via social media (SM), even if the consequences are unintended.^1^

Social media may be defined as web-based and mobile services that allow people to share a connection, monitoring, creating or manipulating text, audio, photos or video, with others.^2^ SM includes social networks (e.g. Facebook, Twitter, WhatsApp and LinkedIn), content-sharing platforms (e.g. YouTube and Instagram), personal and professional blogs, including email, short message service (SMS), electronic journals as well as those published anonymously, Internet discussion forums and the comment sections of websites.^1^

Studies have shown that more than 60% of American physicians use SM to look for medical information and to communicate with peers.^3^ Although no South African data on the use of SM by health professionals are available, it could be assumed that more and more practitioners use social networks, Internet and personal blogs on a daily basis. Social media is seen by today’s practitioners as a work tool because of easy access to smart phones, tablets and other electronic gadgets.^3,4^ The workplace benefits from the use of group-based communication are immense; work interaction groups on WhatsApp are commonly used by professionals to communicate availability for shift work, traffic issues and pictures of patients for obtaining second opinions from colleagues – hence the list is endless.^4^ However, challenges are also associated with some of these platforms because they may not be secure and messages could be sent to wrong recipients, thus compromising privacy and confidentiality.^4^ Social media also presents a challenge of blurring boundaries.^1,4^ Therefore, there is a need for health professionals to acquaint themselves with the recently released guidelines by the HPCSA that deal extensively with SM in terms of its effect on confidentiality, patient and practitioner relationship, and the health professional’s image. Precautionary measures are issued to guide practitioners to protect public.

In conclusion, the health professionals are advised to err on the side of caution when using SM. If professionals are unsure whether it is ethical or not to share particular content via SM, it is best not to do so until advice has been obtained.^1^
